# First case of low-dose umbilical cord blood therapy for pediatric acute respiratory distress syndrome induced by *Pneumocystis carinii* pneumonia

**DOI:** 10.1186/s40001-021-00548-0

**Published:** 2021-08-28

**Authors:** Shuang Liu, Huili Shen, Siyuan Huang, Rong Liu, Dong Qu

**Affiliations:** 1 Pediatric Critical Medicine Department, Children’s Hospital of Capital Institute of Pediatric, No.2 Yabao Road, Chaoyang District, Beijing, 100020 China; 2 Department of Hematology, Children’s Hospital of Capital Institute of Pediatric, Beijing, China

**Keywords:** Umbilical cord blood, Acute respiratory distress syndrome, *Pneumocystis carinii* pneumonia

## Abstract

**Objective:**

This study aimed to present the case of a boy with acute distress syndrome (ARDS) treated with low-dose umbilical cord blood (UCB) therapy and explore the underlying possible mechanism.

**Methods:**

A 7-year-old boy with severe *Pneumocystis carinii *pneumonia and severe ARDS was treated with allogeneic UCB as salvage therapy.

**Results:**

The patient did not improve after being treated with lung protective ventilation, pulmonary surfactant replacement, and extracorporeal membrane oxygenation (ECMO) for 30 days. However, his disease reversed 5 days after allogeneic UCB infusion, and he weaned from ECMO after 7 days of infusion. Bioinformatics confirmed that his Toll-like receptor (TLR) was abnormal before UCB infusion. However, after the infusion, his immune system was activated and repaired, and the TLR4/MyD88/NF-κB signaling pathway was recovered.

**Conclusion:**

Allogenic UCB could treat ARDS by repairing the TLR4/MyD88/NF-κB signaling pathway, thereby achieving stability of the immune system.

## Introduction

Acute respiratory distress syndrome [1] (ARDS) refers to acute progressive respiratory distress and hypoxemia caused by various intrapulmonary and/or extrapulmonary factors. ARDS is a common critical illness with a hospital mortality rate of more than 40% [2]. The pathogenesis of ARDS is mainly related to uncontrolled inflammatory response, dysfunction of the alveolar capillary barrier, abnormal coagulation and fibrinolysis, and imbalance of oxidative stress.

COVID-19 was a global epidemic in early 2020. Studies reported that 67%–85% of patient infected with SARS-CoV-2 had ARDS with a mortality rate of 61.5% [3, 4]. The mortality of severe ARDS was up to 70%. The existing treatment methods were lung protective ventilation strategy, pulmonary surfactant (PS) replacement, prone position, and extracorporeal membrane oxygenation (ECMO). Effective treatment for ARDS pathophysiology is still lacking. Recently mesenchymal stem cells (MSCs) [5] are constantly being explored to treat ARDS because of their exuberant paracrine and direct differentiation. During the epidemic of COVID-19, our center treated a patient with severe ARDS and *Pneumocystis carinii* pneumonia (PCP) with lung protective ventilation, PS replacement, ECMO therapy and allogeneic UCB infusion. The child was evacuated from ECMO and discharged from the hospital. The clinical changes and therapeutic mechanism of UCB were analyzed, hoping to provide a reference for ARDS cell therapy.

## Clinical data and results

A 7-year-old boy was admitted to our center because of a cough for 24 days and shortness of breath for 10 days after exercise. He was diagnosed with nephrotic syndrome 3 years ago and treated with prednisone, cyclosporin, and tacrolimus regularly despite multiple recurrences. He was given rituximab (RTX) two times 2 months before admission. He never received any PCP prophylaxis. After admission, the child had an intermittent fever, hypoxia, and dyspnea with no B cells and 81.13/UL CD4 + T cells being (see Table 1). Computed tomography of the chest indicated diffuse double-lungs patchy ground glass density shadow. The BLAF was positive for PCP polymerase chain reaction. Then, he was treated with cotrimoxazole and carpofol for antibacterial and noninvasive and invasive ventilation for 5 days, respectively, to achieve respiratory support. However, his lung lesions were aggravated, and he had pneumothorax, mediastinal emphysema, and carbon dioxide retention. Severe ARDS (OI = 30) was diagnosed, and he was treated using venovenous ECMO and a high-frequency ventilator.

**Table 1:** Lymphocytes and inflammatory factor changes before and after UCB infusion

Parameters	Before UCB infusion	After UCB infusion	*P* value
CD3 + T-lymphocyte [*M(IQR)*,/μl]	1536.10 (894.08, 1658.14)	1354.46 (1244.18, 1907.08)	0.796
CD4 + T-lymphocyte [*M(IQR)*,/μl]	693.80 (203.59, 822.66)	709.72 (639.38, 925.22)	0.608
CD8 + T-lymphocyte [*M(IQR)*,/μl]	617.61 (376.96, 760.51)	564.36 (1527.14, 925.19)	0.796
CD19 + B-lymphocyte [*M(IQR)*,/μl]	0.35 (0.00, 0.76)	0.00 (0.00, 0.38)	0.590
CD16 + /56 + natural killer cell [*M(IQR)*,/μl]	163.67 (63.62, 177.130	520.45 (397.74, 659.55)	0.020
total lymphocytes [*M(IQR)*,/μl]	1698.64 (963.24, 1837.78)	2186.45 (1932.89, 2486.45)	0.197
TNF-α[*M(IQR)*, pg/ml]	11.45 (9.51, 16.23)	3.8 (3.8, 3.8)	0.018
IL-6[*M(IQR)*, pg/ml]	17.70 (9.71, 32.95)	5.1 (5.1, 5.1)	0.018
IL-8[*M(IQR)*, pg/ml]	28.00 (17.08, 55.10)	1.5 (1.5, 1.5)	0.018
IL-10[*M(IQR)*, pg/ml]	8.78 (5.75, 10.26)	0.4 (0.4, 0.4)	0.018
IL-1β[*M(IQR)*, pg/ml]	5.00 (5.00, 5.63)	0.7 (0.7, 0.7)	0.009
PCT (*mean* ± *SD*, ng/ml)	2.33 ± 3.43	0.21 ± 0.12	0.006

*UCB* umbilical cord blood, *TNF*-*α* tumor necrosis factor-α, *IL* interleukin, *PCT* procalcitonin

**Fig. 1:**
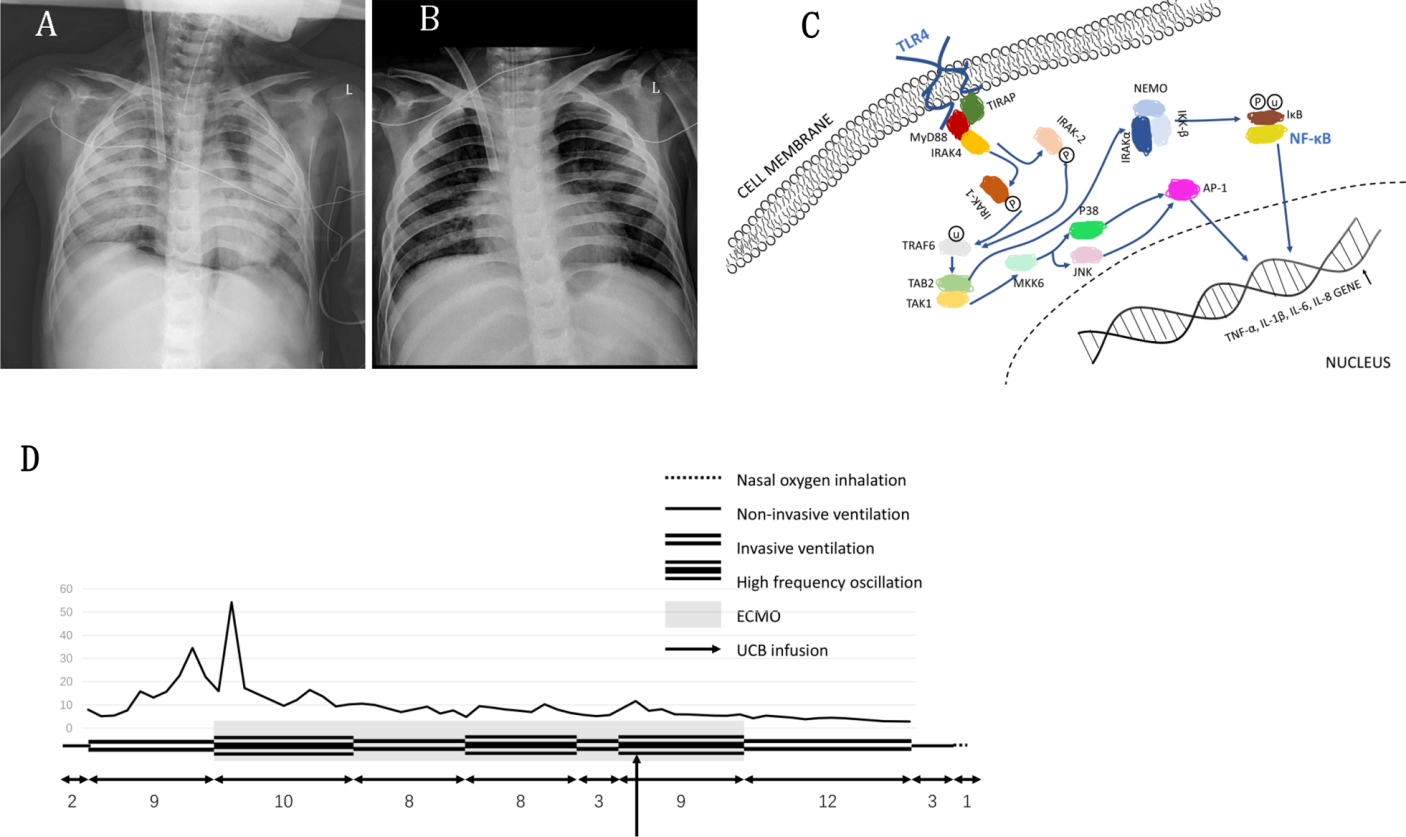
clinical and treatment data of patient.** A** X-ray chest images of patient’s bilateral pulmonary: the day before using umbilical cord blood (UCB) therapy; **B** X-ray chest images of patient’s bilateral pulmonary: the 6th day after using UCB therapy; **C **Toll-like receptor-4(TLR-4)/NF-κB pathway before umbilical stem cells; **D** changes of clinical treatment and OI. NOTE: OI: oxygenation index; ECMO: extracorporeal membrane oxygenation; UCB: umbilical cord blood

After 30 days of full-flow ECMO support, no improvement in lung imaging compared with the previous one was observed (Fig. 1A). At this juncture, after extensive discussions with the family, hospital administration ethics board, and the lawyer, UCB obtained from a healthy newborn was systemically infused through a central venous catheter positioned in the left femoral vein as sympathetic treatment. The blood type of the donor UCB was type A, which was four-sixths consistent with the recipient's HLA, and the A site was not consistent. The total amount of UCB transfused was 4 mL, which contained 2.03 × 10^8^ (0.77 × 10^7^ cells/kg) total nucleated cells and 0.66 × 10^6^ (0.25 × 10^5^ cells/kg) live CD34 cells. The whole input process was divided into three times, and the sequential doses were 2, 1, 1 mL. The ECMO outflow cannula was clamped during each infusion to avoid the infusion of HSCs into the ECMO circuit and maximize the delivery of cells to the pulmonary circulation [6]. The extracorporeal circulation was suspended for 1 min and then the ECMO was resumed for 5 min. At the last operation, the cardiopulmonary bypass was suspended for 2 min. The whole process lasted for 15 min. During the operation, the FiO2 increased to 100%, and the MAP increased by 2 cm H2O using high-frequency ventilator support. The patient's heart rate was maintained at 120–140 beats per minute, and no adverse reactions occurred.

Surprisingly, the lung X-ray examination showed that the condition of the lungs was better than that before on the fifth day after injection (Fig. 1B), and the ECMO condition gradually lowered. On the fifth day, the X-ray indicated that the lung lesions further improved. On the seventh day, the ECMO and high-frequency ventilator were successfully withdrawn. After 4 days of invasive pulmonary support, he had no dyspnea under nasal catheter oxygen. He was discharged from the hospital after 71-day hospitalization. During the 7-month follow-up, his lung function recovered significantly.

Bioinformatics confirmed that the patient’s TLR4/NF-κB pathway was abnormal (Fig. 1C). TLR could not activate MyD88 and IRK4 due to low transcription signals. TRAF6 and RANK did not form tight bound complexes; thus, IKK downstream signals could not be activated. The IRF5 channels were too weak to activate DC chemokines and IL-8, directly affecting the macrophages to clear pathogens in alveolar type I cells. After allogeneic UCB infusion, the patient's condition reversed. Retesting revealed that the immune system was activated and repaired. The TLR4/MyD88/NF-κB signaling pathway was restored to its working capacity (see Table 1).

## Discussion

Allogenic UCB was used in this study for the first time to successfully treat pediatric ARDS. Meanwhile, this study analyzed the mechanism of ARDS at the cellular and molecular levels, and recommended the treatment. Allogeneic UCB treatment could stabilize the immune system and control self-excitation by repairing the TLR4/MyD88/NF-κB signaling pathway.

The patient had nephrotic syndrome with hormone resistance. Moreover, he was treated with prednisone, tacrolimus, and RTX for immunosuppressive therapy. RTX [7] prevents B-cell proliferation and differentiation and depletes B cells [8]. Tacrolimus inhibits T-lymphocyte activation [9]. At the onset of the disease, the patient was depleted of B cells, with low CD4 + T cells. Moreover, he had a PCP infection. Pneumocystis cell wall β-glucan binds to the Toll-like receptor (TLR) on alveolar macrophages, dendritic cells, and lung epithelial cells and activates the CD4 + T cell host. Then it produces inflammatory factors through the NF-κB pathway, enhancing the host adaptive response and clearing PCP [10]. In this study, the patient’s intrinsic cellular immunity was suppressed, CD4 + T cell recruitment and production of IFN-γ [11] decreased, and DC could not stimulate CD4 + T-cell proliferation and polarization effectively [11]. Because of the lack of B cells to regulate T cells, PCP infection was hard to remove because of heavy fungal load. T cells were recovered after stopping tacrolimus which further increased the inflammation of the lung injury. Even with ECMO-supported treatment, the survival rate of patients with non-HIV and PCP was only 8.3%, with a one-third success rate of ECMO offline [3].

Abnormalities in the TLR4/NF-κB signaling pathway were confirmed by genomic and proteomic analyses. TLR4/NF-κB is the main signaling pathway for lung infection [12], ARDS, and various inflammatory responses [13, 14]. Activation of TLR and IL-R could induce dimerization of the aptamer protein MyD88 [15]. MyD88 is a downstream signaling adapter protein, which is essential for cytokine production in TLR ligand reactions [16]. Then, other IRAK1 and IRAK4 interactions to form oligomer complexes and induce TRAF6 dimerization, which leads to IκB-αphosphorylation and degradation [17], de-inhibiting the NF-κB/Rel complex. Pro-inflammatory factors included TNF-α, IL-6, IFN-γ, IL-1, IL-5, and so forth. [18, 19]. The anti-TLR4 monoclonal antibody has been reported to reduce ventilator-induced lung injury in rats by inhibiting MyD88 and NF-κB signaling [20]. The patient could not recover lung function after SMZ combined with caspofungin, respiratory support, and ECMO treatment after 4 weeks. Alveolar lavage still showed a heavy lung inflammatory reaction. The patient could not recover because of his natural immune deficiency and TLR4/MyD88/NF-κB signaling pathway inactivation. The key to the treatment included immune regulation and signaling pathway repair.

With the global epidemic COVID-19 in 2020, the advantages [21] and potential [22] of stem cells to prevent severe COVID-19 pneumonia-induced ARDS were confirmed. Studies proved that MSCs can secrete factors, such as BD-2 [23] through the TLR4/NF-κB pathway, and the production IL-6 and IL-8 [24] factors reduced to alleviate ARDS. UCB is rich in hematopoietic stem cells, MSCs, various types of immune active cells, and their precursor cells (CTL, NK, DC, and Treg) [25]. UCB can be expanded and induced to differentiate into functional mature effector cells, which can quickly complete immune initiation, and regulation and maintain immune response [23]. It plays a very important role in the clinical prevention and treatment of viral infection, elimination of minimal residual disease, suppression of immune rejection, and treatment of autoimmune diseases. UCB could be an effective treatment for the patient discussed in this study theoretically. After UCB infusion, the TLR4/NF-κB signaling pathway was restored, the cytokine levels in vivo returned to normal rapidly, and the number of cellular immune cells gradually returned to normal. When the patient was infected again (8 days after UCB infusion), his cellular immune cells appeared to function and the level of inflammatory factors increased. The safety and efficacy of UCB infusion were verified again.

UCB stem cells are biologically closer to embryonic stem cells, with higher plasticity, faster growth, and greater immune tolerance. Moreover, they can be obtained without invasive treatment in untreated patients. Allogeneic UCB mononuclear cell therapy in ischemic myocardial infarction, stroke, and cerebral palsy in animal experiments and preliminary clinical studies has been shown to improve cardiac and cerebral functions [26]. Autologous UCB infusion in premature infants can reduce the use of ventilator time and oxygenation [27] and treat autism [28]. UCB treatment is safe in premature infants and children, and no serious adverse reactions have been reported.

This study focused on one case of allogeneic UCB therapy for ARDS; large-scale clinical studies are required to validate the results. In addition, the pathogenesis of ARDS and the signaling pathways involved are complex and clinically heterogeneous. The immune regulation of UCB and the mechanism of lung function repair need further basic research. UCB therapy may be a safe and effective way for ARDS treatment.

## Data Availability

The data and material used and/or analyzed of this patient are available from the corresponding author on reasonable request.

## Abbreviations

ARDS: Acute respiratory distress syndrome
ECMO: Extracorporeal membrane oxygenation
MSC: Mesenchymal stem cells
PCP: *Pneumocystis carinii* Pneumonia
PS: Pulmonary surfactant
RTX: Rituximab
TLR: Toll-like receptor
TNC: Total nucleated cells
UCB: Umbilical cord blood
